# Study on the predictive role of bile acid and lymphocyte count in the prognosis of patients with traumatic brain injury: a retrospective analysis

**DOI:** 10.3389/fneur.2025.1601987

**Published:** 2025-11-05

**Authors:** Jie Xu, Yinghua Song, Xiancheng Chen, Ming Chen, Yun Zhao, Zhi Li, Songyang Li, Liang Zhang, Wenkui Yu

**Affiliations:** ^1^Department of Intensive Care Unit, Nanjing Drum Tower Hospital, Clinical College of Nanjing, University of Chinese Medicine, Nanjing, China; ^2^Department of Emergency Intensive Care Unit, Affiliated Xuzhou Municipal Hospital of Xuzhou Medical University, Xuzhou, China; ^3^Department of Emergency Intensive Care Unit, Xuzhou First People’s Hospital, Xuzhou, China; ^4^Department of Pharmaceutical Engineering, Jiangsu Provincial Xuzhou Pharmaceutical Vocational College, Xuzhou, China

**Keywords:** bile acid, lymphocyte count, traumatic brain injury, GCS, retrospective study

## Abstract

**Objectives:**

In patients with traumatic brain injury (TBI), primary traumatic brain injury is often accompanied by gastrointestinal immune dysfunction. Disruption of gut microbiota, abnormal metabolic products of gut microbiota, and immune dysfunction in the body after traumatic brain injury may lead to secondary traumatic brain injury. Bile acids are common metabolites of the gut microbiota in clinical practice, and lymphocyte count is one of the indicators reflecting the clinical immune function of patients. We conducted a retrospective analysis to investigate the value of factors such as bile acids and lymphocyte counts in predicting 30 day mortality in patients with traumatic brain injury.

**Methods:**

This study included 165 patients with TBI and 131 healthy individuals who underwent physical examinations during the same period as the control. The results revealed that compared to the control group, patients with TBI had significantly lower levels of bile acids and lymphocyte counts. Regression analysis reported a correlation between GCS score, bile acid level, lymphocyte count, and 30-day mortality rate in TBI patients. Patients with lower GCS, lower bile acids, and lower lymphocyte counts after TBI have a significantly higher mortality rate during hospitalization.

**Results:**

Compared with healthy individuals undergoing physical examination, the serum bile acid levels of TBI patients were significantly reduced (7.20 ± 2.83 vs. 3.45 ± 1.97 *μ* mol/L, *p* < 0.001). Compared with healthy individuals undergoing physical examination, the lymphocyte count of TBI patients (4.61 ± 1.72 vs. 1.49 ± 0.88 × 10^9^/L, *p* < 0.001) was significantly reduced. Multiple logistic regression analysis of risk factors for 30 day mortality in TBI patients revealed a correlation between bile acids and mortality (OR 0.748 [0.566–0.988]; *p* = 0.041), as well as a positive correlation between lymphopenia and 30 day mortality (OR 0.494 [0.300–0.815]; *p* = 0.006). For TBI patients, bile acid levels, lymphocyte counts, and GCS scores are equally sensitive and specific for predicting 30 day mortality. AUC, Area under the curve. GCS (AUC = 0.7008, *p* = 0.0004), BA (AUC = 0.7274, *p* < 0.0001), LYC (AUC = 0.6678, *p* = 0.0029).

**Conclusion:**

This study provides preliminary clinical evidence for the correlation between bile acid levels, immune function, and mortality in TBI patients. Further research is needed to verify the mechanism of their correlation and provide a theoretical basis for intervening in secondary brain injury after TBI and reducing clinical mortality.

## Introduction

Traumatic brain injury (TBI) is the leading cause of death and disability in patients with trauma (1). It is estimated that approximately 70 million individuals suffer from traumatic brain injury annually (2). The pathological and physiological processes of TBI are complex, and it changes over time, which makes it difficult to cure and gives it a poor prognosis (3). TBI is a complex injury involving the primary injury that occurred at the time of trauma and secondary injury developed hours to months following trauma (4). TBI occurs when the brain parenchyma sustains a physical injury as a consequence of an external application of energy; this causes secondary inflammation to persist for an extended period, including an increase of cerebral edema and breakdown of the blood–brain barrier, metabolic dysfunctions, and oxidative stress, which is not only detrimental to the brain but also can have significant adverse effects on various organ systems, such as respiratory system, cardiovascular system, and gastrointestinal system (5). Clinical and translational laboratory studies have demonstrated the relevance of interactions between the injured brain and distant organs, and complex crosstalk mechanisms between the injured brain and remote organs have been identified (6). The main reasons for changes in intestinal function after TBI may be alteration in visceral perfusion and dysbiosis of gut microbiota. Research has found that TBI may affect the brain-gut axis by altering the central nervous system, intestinal nervous system, gut microbiota, and metabolites (6).

Common gut microbiota metabolites include short-chain fatty acids, tryptophan metabolites, bile acids, etc. Research has found that bile acids significantly improve the central nervous system in neurodegenerative diseases and acute traumatic brain injury (7, 8). There are reports that bile acids and their receptors were associated with TBI and related pathology changes. Experimental evidence suggests that after trauma, bile acid transporters expressed by hypothalamic neurons exhibit abnormal changes. After traumatic brain injury, certain bile acids and analogs can also affect neuroinflammation and cell apoptosis. These results indicate that bile acids play a specific role in the disease progression of TBI (9, 10).

After TBI, peripheral immune cells are recruited into the brain parenchyma, exacerbating the disruption of the brain–blood barrier (BBB) and promoting polarization of microglia/macrophages towards a pro-inflammatory state (11). The blood–brain barrier disruption can simultaneously activate microglia, forming a feedforward loop (12). Through this approach, three active events - immune cell infiltration, blood–brain barrier disruption, and pro-inflammatory microglial/ macrophage status - reinforce each other, forming a vicious cycle that continuously exacerbates brain damage. In animal and human studies, neuroinflammation has been recognized as an essential and manipulable aspect of secondary injury. Because neuroinflammation can be both harmful and beneficial, there is a need to better understand the timing and complexity of the immune response after TBI before developing immunomodulatory therapies (13).

However, current research primarily focuses on animal models of TBI. Compared with experimental studies, no direct clinical evidence exists for changes in bile acid metabolism and immune indicators in TBI patients. In this study, based on existing laboratory evidence, we attempted to conduct a retrospective clinical analysis of TBI patients and healthy individuals to investigate changes in bile acid metabolism and immune indicators after TBI. We also conducted group analysis to explore other factors affecting bile acid levels and immune indicators in TBI patients.

## Methods

### Inclusion criteria

This retrospective study included TBI patients admitted to two medical centers from January 2021 to July 2023 and healthy controls who underwent physical examinations at the medical centers during the same period. TBI was defined by a head Abbreviated Injury Scale (AIS) ≥ 2 or any intracranial hematoma (e.g., cerebral contusion; subarachnoid, subdural, or epidural hemorrhages) seen on head CT scans (14). Inclusion criteria for patients with traumatic brain injury: (1) adults aged 18–75 years old; (2) Meets the diagnostic criteria for traumatic brain injury; (3) The patient received laboratory test results of a standard fasting blood sample upon admission, which should also include indicators such as serum bile acid levels, lymphocyte count, IL-6, and CRP. Exclusion criteria include: (1) patients with combined abdominal injuries, including closed abdominal injuries and open abdominal injuries; (2) History of primary liver, gallbladder, pancreatic, and intestinal obstruction, or history of liver, gallbladder, and gastrointestinal surgery; (3) History of immune system diseases, including immunodeficiency, immunoproliferative diseases, and autoimmune diseases; (4) The patient used steroids or antibiotics 1 month before admission.

The criteria for inclusion in healthy controls are: (1) adults aged 18–75 years old; (2) The patient’s physical examination includes indicators such as serum bile acid levels, lymphocyte count, IL-6, and CRP. Exclusion criteria include: (1) a history of primary liver, gallbladder, pancreatic, or intestinal obstruction, or a history of liver, gallbladder, or gastrointestinal surgery; (2) History of immune system diseases, including immunodeficiency, immunoproliferative diseases, and autoimmune diseases; (3) Those who used steroids or antibiotics 1 month before the physical examination.

### TBI patients stratification

Patients were classified into three subgroups based on TBI severity by the Glasgow Coma Scale (GCS): mild (GCS13-15), moderate (GCS9-12), and severe (GCS ≤ 8) (15). The outcome of TBI patients is a 30 day mortality rate.

### Blood sample details and laboratory tests

We collected clinical data including gender, age, cause of injury, diagnosis, number of days of TBI events, GCS at first admission, whether surgery was performed, tolerance for enteral nutrition support, type of antibiotics, alanine aminotransferase (ALT) levels, aspartate aminotransferase (AST) levels, creatinine levels, total bilirubin, serum bile acid levels, lymphocyte count, and IL-6. This article has no traceable personal information, and the study meets the ethical standards of the 1964 Helsinki Declaration and subsequent amendments. Our Medical Center Ethics Committee approved this study with approval number xylyl[2024]047.

This study established strict inclusion criteria for blood sample collection to obtain reliable data such as bile acid, lymphocyte count, and IL-6. Our hospital’s testing center obtains the test results of the included patients to prevent deviations between different laboratories.

All blood samples from patients with TBI were collected within 8 h after admission. This will ensure that patients are most stable before any invasive treatment. Specifically, laboratory testing should cover at least blood routine, liver function, kidney function, Lactic acid, serum bile acids(all are serum total bile acids), and cytokines. Healthy controls, all fasting for 8 h, testing blood routine, liver and kidney function, serum bile acids (all are serum total bile acids), cytokines, and coagulation function.

Bile acid determination: Enzyme cycling method was used to catalyze the oxidation reaction of bile acids using 3α- hydroxysteroid dehydrogenase (3α- HSD), accompanied by the conversion of NAD^+^ → NADH. The bile acid concentration was calculated by detecting the absorbance change of NADH at 340 nm. Our inspection center was responsible for quality control and intra batch/inter batch variability management.

Health control conditions: The control group was required to fast for 8 h before blood draw, and all serum samples were sent to the testing center for testing and quality control using the same method.

### Statistical analysis

Statistical analysis was conducted using Stata 17, R software package (version 4.3.2), and SPSS version 21.0. Continuous variables are given in the form of mean and standard deviation. Use R language to measure the Youden index and critical value of biomarkers. The following numbers and percentages represent categorical variables. To compare continuous variables, perform a student t-test (if normally distributed) or a Mann Whitney U test (if not normally distributed), and variance inflation factor (VIF) values were calculated in the process to rule out the possible impact of multi-collinearity. Compare the categorical variables of chi-square test. Perform linear regression analysis to determine the impact of TBI mortality and VIF. *p* < 0.05 is considered statistically significant.

## Results

### Patient inclusion and characteristics

**Figure 1:**
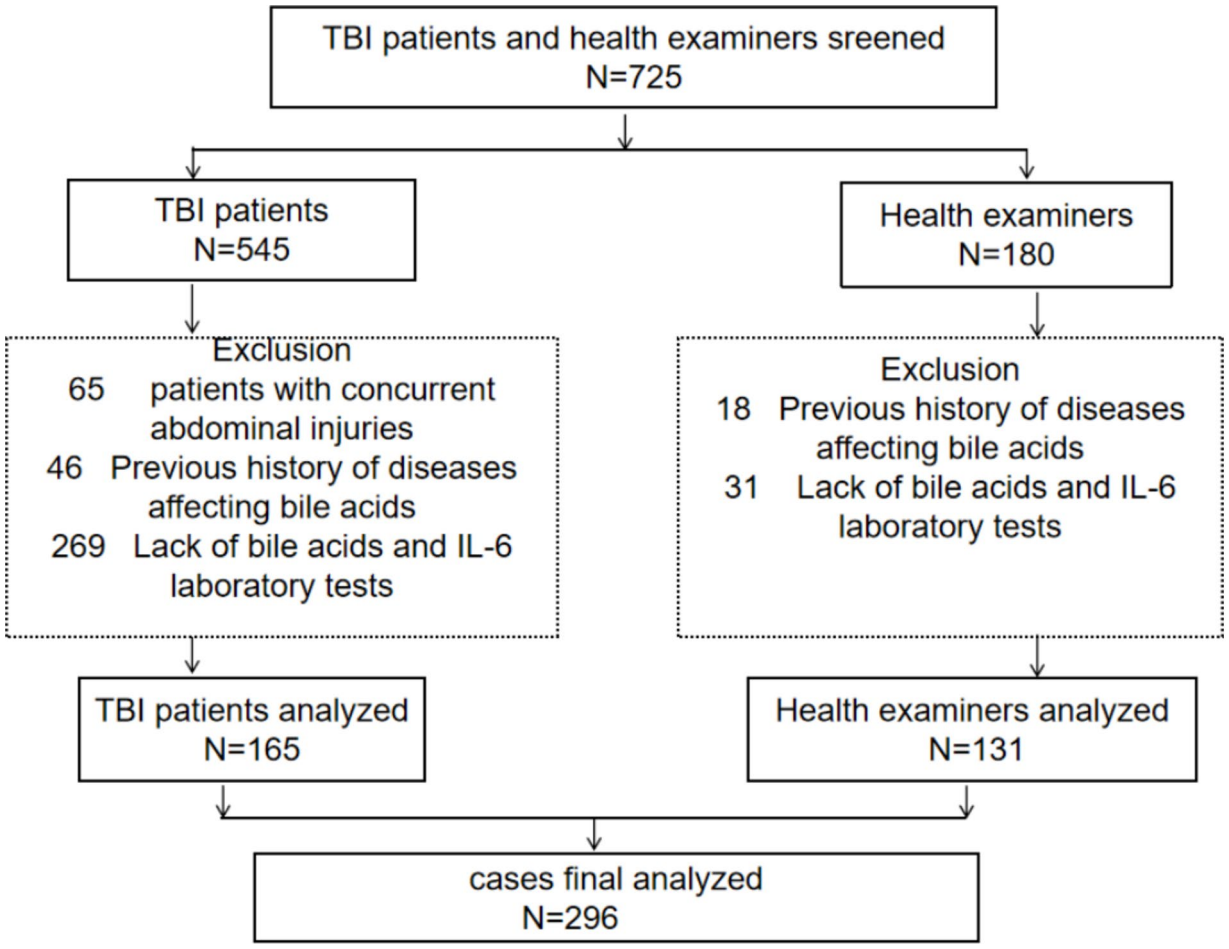
Schematic diagram illustrating detailed inclusion of traumatic brain injury patients.

Figure 1 shows the flowchart of the case collection process. In the initial search process, we collected 545 patients with traumatic brain injury and 180 healthy examinees from the same period. We reviewed the titles and abstracts of 306 studies, and excluded 380 patients with traumatic brain injury for the following reasons: accompanied by abdominal injury (*n* = 65); History of liver and gastrointestinal diseases in the past (*n* = 46); Individuals lacking indicators such as bile acids and IL-6 (*n* = 269). 49 cases were excluded from the health examination due to the following reasons: previous medical history of liver, gastrointestinal tract, etc. (*n* = 18); Individuals lacking indicators such as bile acids and IL-6 (*n* = 31). Therefore, 296 eligible studies were ultimately included, including 165 patients with traumatic brain injury and 131 healthy individuals undergoing physical examinations. Figure 1 shows the complete case collection strategy and flowchart.

Among the 296 cases in this study, 165 (55.7%) were TBI patients, and 131 (44.3%) were healthy individuals undergoing physical examinations. The average age of 296 cases was 52.7 ± 18.0 years, and 181 (61.1%) patients were male. Upon admission, the GCS of TBI patients was 10.2 ± 4.06, and the time from injury to admission was 8.2 ± 3.75 h. The causes of injury included 87 car accidents, 53 high fall injuries, 20 heavy object injuries, and 5 falls. TBI patients included 115 cases of cerebral contusion and laceration, 45 cases of cerebral concussion, 39 cases of combined traumatic subarachnoid hemorrhage, 36 cases of combined epidural and subdural hematoma, and 75 cases of combined skull fractures. Forty-two patients with TBI underwent craniotomy hematoma removal, and 37 patients underwent drilling and drainage surgery. Of all the included cases, 51 had a history of hypertension, 47 had a history of diabetes, 22 had a history of heart disease, and 19 had a history of chronic obstructive pulmonary disease(COPD). The detailed basic features are shown in Table 1.

**Table 1:** Demographic population of traumatic brain injury.

Characteristics	Value
Numbers	296
Male percentage	61.10%
Age(years)	52.7 ± 18.0
Serum bile acid, mean ± SD (umol/L)	4.3 ± 2.40
Lymphocyte count	3.45 ± 1.36
IL-6	1.76 ± 1.18
CRP	28.54 ± 11.90
Total bilirubin, mean ± SD (umol/L)	9.43 ± 3.15
ALT, mean ± SD (u/L)	27.58 ± 16.43
AST, mean ± SD (u/L)	25.50 ± 14.08
Creatinine, mean ± SD (umol/L)	68.91 ± 16.52
GCS, mean ± SD	10.2 ± 4.06
Medical history (other than exclusion criteria)	
Hypertension	51
Diabetes	47
Heart disease	22
COPD	19
TBI, *n* (%)	
Cerebral contusion	115
Cerebral concussion	45
Traumatic subarachnoid hemorrhage	39
Traumatic epidural and/or subdural hematoma	36
Skull fracture	75
Craniotomy hematoma removal surgery	42
Drilling drainage surgery	37
Time from injury to admission (h)	8.2 ± 3.75
Causes of injury	
Car accidents	87
High fall injuries	53
Heavy object injuries	20
Falls	5

IL-6, Interleukin-6; CRP, C-reactive protein; ALT, alanine aminotransferase; AST, aspartate aminotransferase; GCS, Glasgow Coma Scale; COPD, chronic obstructive pulmonary disease; TBI, traumatic brain injury.

### Differences of characteristics between TBI patients with physical examines

There was no significant difference between the brain injury group and the health examination group in terms of age, gender, hypertension, diabetes, heart disease, and COPD (*p* < 0.05). Compared with healthy individuals undergoing physical examinations, TBI patients’ serum bile acid levels (3.45 ± 1.97 vs. 7.20 ± 2.83 *μ* mol/L, *p* < 0.001) were significantly lower, and the difference was statistically significant. Compared with healthy individuals undergoing physical examinations, the lymphocyte counts of TBI patients (1.49 ± 0.88 vs. 4.61 ± 1.72 × 109/L, *p* < 0.001) were significantly lower, and the difference was statistically significant. Compared with healthy individuals undergoing physical examinations, the IL-6 levels in TBI patients (10.95 ± 1.14 vs. 1.57 ± 1.28 pg/mL, *p* < 0.001) were significantly increased, and the difference was statistically significant. Compared with healthy individuals undergoing physical examinations, the CRP levels in TBI patients (34.48 ± 10.52 vs. 12.63 ± 11.58 mg/L, *p* < 0.001) were significantly increased, and the difference was statistically significant. The total bilirubin, ALT, AST, and blood creatinine of the two groups of patients were similar, and there were no significant differences. The differences of characteristics between TBI patients and physical examinees are shown in Table 2.

**Table 2:** Differences of characteristics between TBI patients and physical examinees.

Characteristic	TBI	Physical examinations	*p*-value
Numbers	165	131	
Gender (male)	96 (58.1%)	83 (63.9%)	0.743
Age	55.2 ± 16.7	50.8 ± 19.6	0.667
Hypertension	29	23	0.23
Diabetes	22	25	0.146
Heart disease	9	13	1.002
COPD	10	9	0.328
Time from injury to admission(h)	8.2 ± 3.75	-	
GCS, mean ± SD	10.2 ± 4.06	15.0 ± 0.00	<0.001
Serum bile acid, mean ± SD (umol/L)	3.45 ± 1.97	7.20 ± 2.83	<0.001
Lymphocyte count(10^9^/L)	1.49 ± 0.88	4.61 ± 1.72	<0.001
IL-6 (pg/ml)	10.95 ± 1.14	1.57 ± 1.28	<0.001
CRP (mg/L)	34.48 ± 10.52	12.63 ± 11.58	<0.001
Total bilirubin, mean ± SD (umol/L)	10.45 ± 3.71	8.49 ± 2.44	0.572
ALT, mean ± SD (U/L)	28.34 ± 20.37	25.65 ± 15.28	1.333
AST, mean ± SD (U/L)	23.62 ± 12.59	27.80 ± 16.64	0.532
Creatinine, mean ± SD (umol/L)	64.78 ± 19.57	70.63 ± 15.47	0.361

COPD, chronic obstructive pulmonary disease; GCS, Glasgow Coma Scale; IL-6, Interleukin-6; CRP, C-reactive protein; ALT, alanine aminotransferase; AST, aspartate aminotransferase.

### Subgroup analyses

Subgroup analyses of patients with traumatic brain injury found that, with whether the patient died within 30 days of admission as the dependent variable, GCS score, PCO2, TBIL, BA, Cr, LYC, IL-6, CRP, and other indicators as independent variables were included in the multivariate logistic regression analysis. The results showed that GCS score, total bile acid, and lymphocyte count were independent risk factors for 30-day mortality in patients with traumatic brain injury (*p* < 0.05). The differences are shown in Table 3.

**Table 3:** Multivariate logistic regression analysis of risk factors for 30-day mortality in TBI patients.

Variable	Odds ratio	Std. err.	z	p	95% conf.interval
GCS	0.796	0.89	−2.04	**0.042**	0.640 to 0.991
PCO2	0.934	0.04	−0.65	0.516	0.899 to 1.055
TBIL	1.024	0.052	0.47	0.641	0.927 to 1.132
BA	0.748	0.106	−2.04	**0.041**	0.566 to 0.988
Cr	1.000	0.008	0.36	0.719	0.987 to 1.019
LYC	0.494	0.126	−2.76	**0.006**	0.300 to 0.815
IL-6	0.997	0.056	−0.05	0.963	0.893 to 1.114
CRP	1.020	0.012	1.69	0.09	0.997 to 1.043

GCS, Glasgow Coma Scale; PCO2,partial pressure of carbon dioxide; TBIL, total bilirubin; BA, bile acid; Cr, Creatinine; LYC, lymphocyte count; IL-6, Interleukin-6; CRP, C-reactive protein. Bold font indicates a *P*-value less than 0.05.

### Nomogram for multiple factor regression analysis in TBI patients

**Figure 2:**
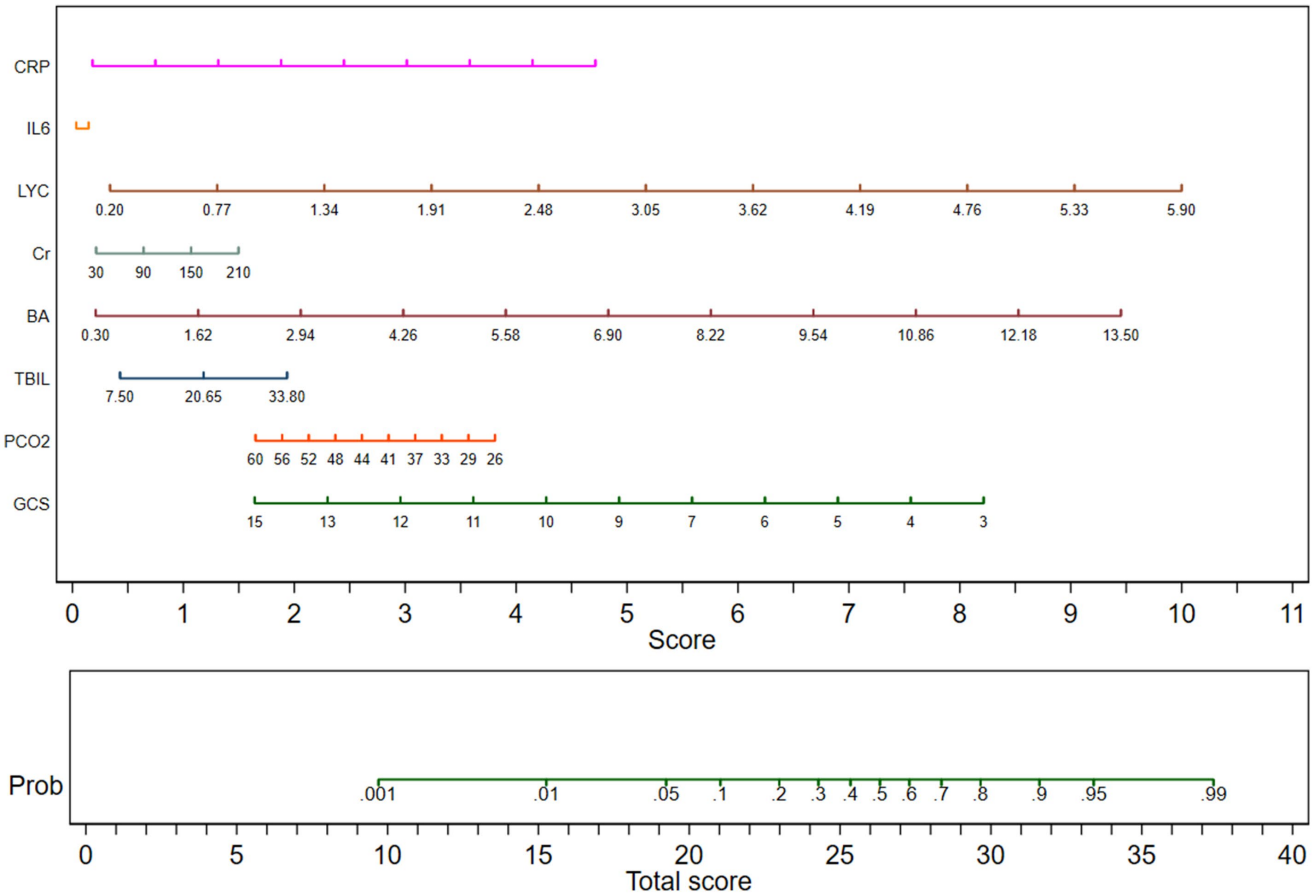
Column chart for multiple factor regression analysis in TBI patients. GCS, Glasgow Coma Scale; PCO2, partial pressure of carbon dioxide; TBIL, total bilirubin; BA, bile acid; Cr, Creatinine; LYC, lymphocyte count; IL-6, Interleukin-6; CRP, C-reactive protein.

Subgroup analysis was conducted on patients with traumatic brain injury, and multiple logistic regression analysis was included. The nomogram displayed the GCS, PCO2, total bilirubin, total bile acid, blood creatinine, lymphocyte count, IL-6, CRP with TBI patients. The nomogram showed that GCS, BA and LYC are negatively correlated with the 30 day mortality rate of TBI patients. The length of the moderate line in the column chart could intuitively reflect the relative importance of the variable to the prediction results, and the longer the line segment, the greater the impact of the variable on the results. Indicating that besides GCS, BA and LYC have good predictive value for the prognosis of TBI patients. The predicted values are shown in Figure 2.

### Net reclassification improvement and integrated discrimination improvement for 30-day mortality among physical functions

To determine the accuracy of predicting 30 day mortality, the incremental predictive value of lymphocyte count, serum bile acid, and other physiological functional indicators on the basic clinical model (including age, gender, and GCS score) was compared. The groups were compared by comparing Model 1 (basic clinical model: age, gender, GCS at admission), Model 2 (Model 1 + lymphocyte count), Model 3 (Model 1 + total bilirubin), Model 4 (Model 1 + serum bile acids), Model 5 (Model 1 + serum creatinine), Model 6 (Model 1 + C-reactive protein), and Model 7 (Model 1 + interleukin-6). When lymphocyte count is combined with basic clinical models, both net weight classification improvement index (NRI = 0.421, 95% CI: 0.124–0.741, *p* = 0.004) and comprehensive discriminant improvement index (IDI = 0.335, 95% CI: 0.317–0.803, *p* = 0.004) show significant improvement in predictive ability; When serum bile acids are used in combination with basic clinical models, both the net weight classification improvement index (NRI = 0.365, 95% CI: 0.057–0.809, *p* = 0.028) and the comprehensive discriminant improvement index (IDI = 0.441, 95% CI: 0.008–0.045, *p* = 0.002) show significant improvement in predictive ability. In addition, when lymphocyte count and serum bile acid were combined with clinical models, the net weight classification improvement index (NRI = 0.083, 95% CI: 0.156–0.867, *p* = 0.009) and comprehensive discrimination improvement index (IDI = 0.438, 95% CI: 0.021–0.076, *p* = 0.007) both showed significant improvement in predictive ability. The NRI and IDI are shown in Table 4.

**Table 4:** Net reclassification improvement and integrated discrimination improvement for 30-day mortality among physical functions.

	NRI	95%CI	*P*-value	IDI	95%CI	*P*-value
Clinical model	Reference			Reference		
Clinical model + LYC	0.421	0.124 to 0.741	0.004	0.335	0.317 to 0.803	0.004
Clinical model + BA	0.365	0.057 to 0.809	0.028	0.441	0.008 to 0.045	0.002
Clinical model + TBIL	0.176	−0.072 to 0.663	0.208	0.029	0.395 to 0.732	0.667
Clinical model + Cr	0.119	0.043 to 0.990	0.168	0.035	0.459 to 0.719	0.706
Clinical model + CRP	0.021	−0.046 to 0.775	0.500	0.142	0.506 to 0.866	0.504
Clinical model + IL-6	0.295	−0.054 to 0.663	0.098	0.079	0.101 to 0.244	0.832
Clinical model + LYC + BA	0.083	0.156 to 0.867	0.009	0.438	0.021 to 0.076	0.007

Clinical model: age, male, Glasgow Coma Scale; PCO2, partial pressure of carbon dioxide; TBIL, total bilirubin; BA, bile acid; Cr, Creatinine; LYC, lymphocyte count; IL-6, Interleukin-6; CRP, C-reactive protein.

### Multivariate logistic regression analysis and collinearity diagnosis of 30 day mortality in patients with TBI

To explore the potential impact of other factors on bile acid levels and lymphocyte counts in patients with TBI, we also conducted subgroup analysis using regression methods. The data showed that GCS was negatively correlated with 30 day mortality (*p* < 0.05), while lymphocyte count and bile acid levels were positively correlated with 30 day mortality (*p* < 0.05). According to the 30 day mortality rate, we divided patients with TBI into two groups and analyzed the gender, age, GCS, PCO2, total bilirubin, bile acids, creatinine, lymphocyte count, IL-6, and CRP. The results showed no significant collinearity among these factors (VIF < 5), indicating that these factors are independent of each other when predicting 30 day mortality. VIF < 5: No significant collinearity. 5 ≤ VIF < 10: Moderate collinearity, should be interpreted with caution. VIF ≥ 10: Severe collinearity, variables need to be deleted or merged. The differences are shown in Table 5.

**Table 5:** Multivariate logistic regression analysis and collinearity diagnosis of 30 day mortality in patients with TBI.

Factors	β	OR (95%CI)	P	VIF
Gender	0.554	0.467 to 0.891	0.660	1.723
Age	0.029	0.003 to 0.064	0.163	1.088
GCS	−0.073	0.640 to 0.991	0.042	1.790
PCO2	2.075	0.899 to 1.055	0.516	4.332
TBIL	−1.558	0.927 to 1.132	0.641	2.465
BA	0.085	0.566 to 0.988	0.041	1.196
Cr	0.026	0.087 to 1.019	0.719	2.660
LYC	0.572	0.300 to 0.815	0.006	1.767
IL-6	0.928	0.893 to 1.114	0.963	1.338
CRP	0.557	0.997 to 1.043	0.090	3.545

Clinical model: age, male, Glasgow Coma Scale; PCO2,partial pressure of carbon dioxide; TBIL, total bilirubin; BA, bile acid; Cr, Creatinine; LYC, lymphocyte count; IL-6, Interleukin-6; CRP, C-reactive protein.

### Cutoff values and Youden Index of blood bile acid level, and lymphocyte count for 30-day mortality in TBI patients

For TBI patients, Cutoff values and Youden Index of LYC and BA are shown in Table 6.

**Table 6:** Cutoff values and Youden Index of blood bile acid level, and lymphocyte count.

Biomarker	Cutoff values	Sensitivity (%)	Specificity (%)	Youden Index (J)	AUC (95%CI)	*p* value
LYC	<0.75 × 10^9^	70.5	67.7	0.38	0.67 (0.61–0.75)	<0.001
BA	<5.5umol/L	68.4	72.3	0.45	0.73 (0.67–0.79)	<0.003

LYC, lymphocyte count; BA, bile acid.

### ROC curve of GCS score, blood bile acid level, and lymphocyte count for 30-day mortality in TBI patients

**Figure 3:**
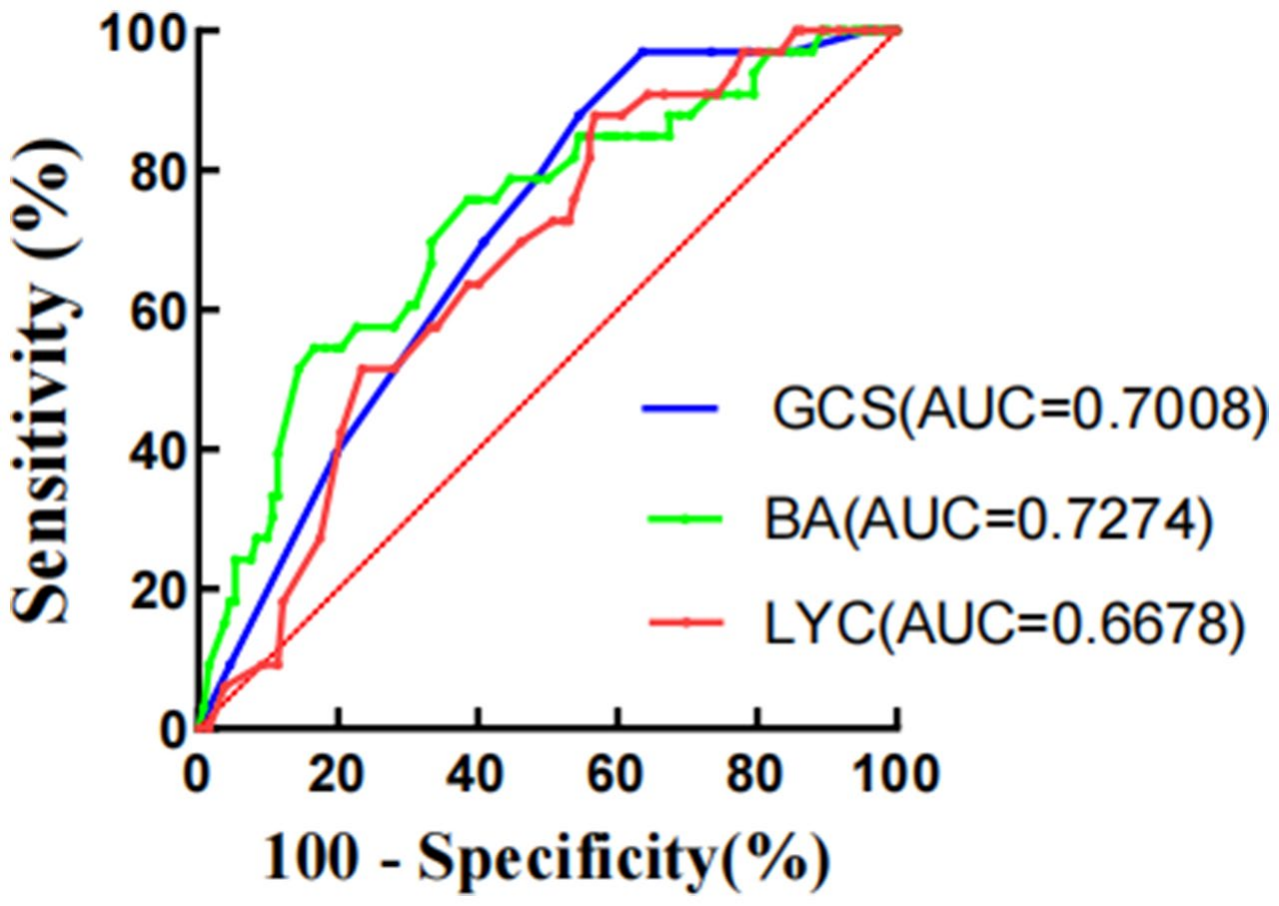
ROC curves of GCS, BA, and LYC in TBI patients.

For TBI patients, BA, LYC, and GCS scores are equally sensitive and specific for predicting 30 day mortality. AUC, Area under the curve, GCS(AUC = 0.7008,*p* = 0.0004), BA(AUC = 0.7274,*p* < 0.0001), LYC(AUC = 0.6678,*p* = 0.0029). The ROC curves of GCS, BA, and LYC in TBI patients is shown in Figure 3.

## Discussion

Brain injury is an acute biomechanical event characterized by multiple pathological and physiological processes that develop over time. TBI patients have lesions characterized by white matter degeneration, neuronal loss, protein misfolding, and persistent neuroinflammation (16). Over time, direct mechanical damage to brain tissue can also lead to secondary brain injury, in which immune disorders and inflammatory responses play essential roles. Catecholamine storm is a common phenomenon that affects the immune system after TBI. There is a close relationship between cytokine networks, systemic inflammatory response syndrome, and immune response. The inflammatory reaction of TBI includes the production of local cytokines and chemokines in the brain, activation of endothelial cells, activation of microglia, and migration of neutrophils, lymphocytes, and monocytes to the site of brain injury. The close relationship between neuropathology and inflammation has been documented. Experimental studies also indicate that specific inhibition of inflammatory responses may protect damaged tissues in the early stages after injury but can damage the brain in the chronic stage. Therefore, treatment strategies should aim to regulate the inflammatory response rather than inhibit it (17).

Secondary injury is a process that occurs continuously for several minutes to years after the initial injury. A cascade of metabolic changes, neurochemical reactions, and cellular and molecular events from the primary injury causes secondary injury. These mechanisms ultimately lead to brain cell death, plasticity changes, tissue damage, and tissue atrophy (18). Some biochemical changes that lead to secondary injury include disrupted cellular calcium homeostasis, glutamate excitotoxicity, mitochondrial dysfunction, increased free radical production, inflammation, increased lipid peroxidation, apoptosis, diffuse axonal injury (DAI), and blood–brain barrier disruption. Of note, all the above-listed factors can be directly or indirectly linked to neuroinflammation, and such inflammation has been implicated in both the early and chronic components of TBI-induced neuropathology (11). There have been reports of increased systemic immune cells in the brain parenchyma after traumatic brain injury in both human and animal models, and these release inflammatory mediators and guide glial and immune cells to the injury site (19). A study found that the biomarker level of plasma Homocysteine (HCY), Serum CRP, matrix metalloproteinase-9 (MMP9), E-selectin (SELE), and P-selectin (SELP)were associated with clinical outcome in patients with ICH in the north Indian population (20). Some studies have shown that lymphopenia of TBI patients on admission is related to the poor prognosis of patients, of which lymphopenia is related to increased mortality, increased incidence rate of pneumonia and urinary tract infection, and prolonged hospitalization (21), which is consistent with our study.

After trauma, the body can undergo complex host reactions, disrupt the dynamic balance of the immune system, and experience immune dysfunction. In recent years, multiple studies have reported that bile acid receptors or transporters, such as TGR5 and apical sodium-dependent bile acid (ASBT) transporter, were involved in the pathological progress after brain injury in animal models (22). Studies had found that Bile acids could promote the TGR5-STAT3-A20 pathway and inhibit the production of inflammatory cytokines mediated by NF-κB and MAPK to prevent LPS induced sepsis (23). Indeed, circulating bile acids are better predictors of sepsis-related mortality than liver function parameters, such as bilirubin (24).

As the most important immune organ in the human body, research has shown that in the context of central nervous system diseases, including traumatic brain injury, changes in the gut microbiota population and its metabolites may occur and may further develop due to the progression of the injury (25). Primary bile acids are synthesized in the liver and then secreted into the intestine. Under the action of gut microbiota, they are converted into secondary bile acids and participate in the digestion and absorption of lipids and fat-soluble vitamins in the diet. Bile acids are closely related to intestinal immunity. Studies have found that intestinal bacteria modify bile acids in the intestine into immune regulatory molecules. The modified bile acids activate two types of immune cells: regulatory T cells (Tregs) and effector helper T cells, especially Th17, which regulate immune responses by inhibiting or promoting inflammation (26). Casper’s experiment has shown that gut microbiota and diet work together to alter bile acids, which in turn affect the Treg levels in mice’s colon. They also indicated that lower levels of Treg cells due to a lack of bile acids or bile acid sensors make animals more susceptible to inflammatory colitis (27). In a retrospective clinical study, compared with patients with trigeminal neuralgia/facial spasm. The serum bile acid levels in patients with TBI were about two-thirds of those in the control group. Subgroup analysis was also conducted on TBI patients, and the results showed that antibiotics, serum creatinine, and triglycerides were associated with changes in serum bile acid levels (28).

In this study, we elucidated the changes in bile acid levels and lymphocyte counts after traumatic brain injury, mainly manifested as a significant decrease in serum bile acid levels and lymphocyte counts upon admission compared to healthy individuals undergoing physical examination without traumatic brain injury. At the same time, further regression analysis was conducted on the subgroups of TBI patients. In addition to GCS scores, changes in bile acid levels and lymphocyte counts in TBI patients were negatively correlated with patient mortality, indicating a correlation between gut microbiota metabolites and intestinal immune function in patients with traumatic brain injury and prognosis. For TBI patients, BA, LYC, and GCS scores are equally sensitive and specific for predicting 30 day mortality, AUC, area under the curve. We believe that after TBI, patients may experience abnormal bile acid metabolism and decreased intestinal immune function, which may promote the development of secondary brain injury and lead to a significant increase in the 30-day mortality rate of TBI patients. Due to the retrospective design, the number of patients included in this study is limited. Only relevant indicators at admission are included for TBI patients, without paying attention to the dynamic changes of indicators such as bile acid, lymphocyte count, liver function, kidney function, GCS score, partial pressure of carbon dioxide, C-reactive protein, IL-6, etc. This makes it difficult to determine whether the prognosis of TBI patients is correlated with the dynamic changes of these indicators. Given these limitations, larger-scale research will be needed to determine the biological roles of gut immune functions such as bile acid levels and lymphocyte counts in TBI disease and their interactions with other physiological progression.

Regardless, there may be some correlation between the bile acid levels, lymphocyte counts, and prognosis of TBI patients, which deserves further research and provides a new method for studying secondary traumatic brain injury after TBI from the perspective of intestinal immune regulation.

## Conclusion

Early detection of hospital complications and mortality rates is crucial for patient management. Here, we identify the bile acid levels and lymphocyte counts of TBI patients upon admission as a potential predictive tool. Our research found a correlation between bile acid levels, decreased lymphocyte counts, and 30 day mortality at admission, which has clinical value similar to GCS. Our research findings suggest that all TBI patients should pay attention to their gut microbiota, metabolic products, and gut immunity. This will help clinicians identify high-risk patients with adverse consequences and enable them to better provide them and their families with expected clinical hospitalization recommendations. Prospective observational studies from the perspective of the brain gut axis in the future will help confirm our results.

## Data Availability

The original contributions presented in the study are included in the article/supplementary material, further inquiries can be directed to the corresponding author.
